# ERGA-BGE reference genome of *Lewinskya acuminata, *a common epiphytic Mediterranean moss with disjunct populations in California and Ethiopia

**DOI:** 10.12688/openreseurope.21670.1

**Published:** 2025-11-24

**Authors:** Pablo Aguado-Ramsay, Francisco Lara, Isabel Draper, Maria Conejero, Astrid Böhne, Rita Monteiro, Thomas Marcussen, Torsten H. Struck, Rebekah A. Oomen, Genomescope Sequencing Team, Manon Angel, Jean-Marc Barbance, Julie Batisse, Odette Beluche, Laurie Bertrand, Elodie Brun, Maria Dubois, Corinne Dumont, Barbara Estrada, Thomas Guerin, Zineb El Hajji, Sandrine Lebled, Patricia Lenoble, Claudine Louesse, Ghislaine Magdelenat, Eric Mahieu, Claire Milani, Sophie Oztas, Marine Paillard, Emilie Payen, Emanuelle Petit, Murielle Ronsin, Benoit Vacherie, Alice Moussy, Corinne Cruaud, Karine Labadie, Lola Demirdjian, Emilie Téodori, Patrick Wincker, Pedro H. Oliveira, Jean-Marc Aury, Chiara Bortoluzzi

**Affiliations:** 1 Departamento de Biología, Facultad de Ciencias, C. Darwin 2, Universidad Autónoma de Madrid, Madrid, 28049, Spain; 2 Centro de Investigación en Biodiversidad y Cambio Global, C. Darwin 2, Universidad Autónoma de Madrid, Madrid, 28049, Spain; 3 Department of Biodiversity and Evolutionary Biology, Museo Nacional de Ciencias Naturales (MNCN-CSIC), Madrid, Spain; 4 Leibniz Institute for the Analysis of Biodiversity Change, Museum Koenig Bonn, Bonn, 53113, Germany; 5 University of Oslo, Natural History Museum, London, England, UK; 6 University of Oslo, Centre for Ecological & Evolutionary Synthesis, Oslo, Norway; 7 Department of Biological Sciences, University of New Brunswick Saint John, Saint John, Canada; 8 Tjärnö Marine Laboratory, University of Gothenburg, Gothenburg, Sweden; 9 Centre for Coastal Research, University of Agder, Kristiansand, Norway; 10 Genoscope, CEA, CNRS, Univ Evry, Université Paris-Saclay, Institut François Jacob, Evry, 91057, France; 11 Genoscope, Institut François Jacob, CEA, CNRS, Univ Evry, Université Paris-Saclay, Génomique Métabolique, Evry, 91057, France; 12 SIB Swiss Institute of Bioinformatics, Amphipôle, Quartier UNIL-Sorge, Lausanne, 1015, Switzerland

**Keywords:** Lewinskya acuminata, genome assembly, European Reference Genome Atlas, Biodiversity Genomics Europe, Earth Biogenome Project, Bryophyta, moss, Orthotrichaceae

## Abstract

The reference genome of *Lewinskya acuminata* (H. Philib.) F. Lara, Garilleti & Goffinet will enable phylogenomic, biogeographic, and evolutionary studies within the *Orthotrichaceae* and related bryophyte lineages at a depth previously inaccessible. This species of moss is among the most representative of the Mediterranean epiphytic communities and can be readily identified by its long-acuminate leaves, fusiform capsules with a vestigial exostome, a well-developed endostome of six broad segments, and a dark, puckered peristome mouth when dry. The entirety of the genome sequence was assembled into 6 contiguous chromosomal pseudomolecules, 1 mitochondrial genome, and 2 plastid genomes. This chromosome-level assembly encompasses 0.25 Gb, composed of 51 contigs and 13 scaffolds, with contig and scaffold N50 values of 11.5 Mb and 40.8 Mb, respectively.

## Introduction

*Lewinskya acuminata* was originally described as *Orthotrichum acuminatum* H. Philib. ( Philibert, 1881) and was subsequently transferred to the newly segregated genus *Lewinskya* after the recent division of *Orthotrichum*, which separated the monoicous species with superficial stomata ( Lara *et al.*, 2016). It is an epiphytic moss of the family Orthotrichaceae, readily distinguished by its long-acuminate leaves and characteristic fusiform capsules with a vestigial exostome, a developed endostome of six broad segments, and a dark, puckered mouth when dry. This configuration of the peristome and capsules allows for the hygrocastic release of spores (in conditions of high atmospheric humidity and moss hydration), while most of its congeners do so in dry conditions, xerocastically ( Lara *et al.*, 1999). The species is widespread across the Mediterranean Basin, occurring on most major islands and mainland territories of both Europe and North Africa, from Anatolia (Turkey) to the Iberian Peninsula, Morocco, and Macaronesia ( Calleja *et al.*, 2020). In recent decades, it has also been recorded in scattered localities across northwestern and central Europe ( Müller, 2019). Furthermore, disjunct populations have been discovered in California and Ethiopia ( Calleja *et al.*, 2020; Vigalondo *et al.*, 2016). This moss is one of the most representative species within Mediterranean epiphytic communities ( Lara & Garilleti, 2014), contributing to local biodiversity in a significant part of the Western Palearctic region. This species is autoicous and karyotype analyses of most species within the genus have revealed a haploid chromosome number of six ( Rice *et al.*, 2015).

*Lewinskya acuminata* has been assessed for the IUCN Red List of Threatened Species (Europe assessment) in 2018 and is listed as *Least Concern*. Other conservation categories have been proposed for some European territories: Vulnerable (VU) in Great Britain; Data Deficient (DD) in Madeira, Montenegro, and Slovenia; and Near Threatened (NT) in Sicily ( Hodgetts & Lockhart, 2020).

The generation of a reference genome for *Lewinskya acuminata* will allow for in-depth studies of evolutionary processes, genetic diversity, and speciation patterns within Orthotrichaceae, and will contribute to the conservation and taxonomic assessment of both described and yet-undescribed species ( Aguado-Ramsay *et al.*, 2024; Matanov *et al.*, 2025). *Lewinskya* is a cosmopolitan genus with approximately 80 recognized species, several of which have emerged from recent taxonomic studies (e.g., Lara *et al.*, 2024; Lara *et al.*, 2025; Vigalondo *et al.*, 2020). Several other complexes from different continents are being re-evaluated through integrative taxonomy, for which the reference genome is likely to be extremely valuable.

The generation of this reference resource was coordinated by the European Reference Genome Atlas (ERGA) initiative’s Biodiversity Genomics Europe (BGE) project, supporting ERGA’s aim of promoting transnational cooperation to promote advances in the application of genomics technologies to protect and restore biodiversity ( Mazzoni *et al.*, 2023).

## Materials & methods

ERGA's sequencing strategy includes Oxford Nanopore Technology (ONT) and/or Pacific Biosciences (PacBio) for long-read sequencing, along with Hi-C sequencing for chromosomal architecture, Illumina Paired-End (PE) for polishing (i.e. recommended for ONT-only assemblies), and RNA sequencing for transcriptomic profiling, to facilitate genome assembly and annotation.

### Sample and sampling information

On 31 January 2024, Pablo Aguado-Ramsay and Francisco Lara sampled one specimen of *Lewinskya acuminata* (hermaphrodite monoecious). The specimen was determined based on morphology using Lara & Garilleti (2014) and it was identified by Pablo Aguado-Ramsay and Francisco Lara in Hoyo de Manzanares, Madrid, Spain. The biological material collected in Spain, and used to generate digital sequences, was retrieved from wildlife taxa regulated by the Spanish Royal Decree 124/2017 ( https://www.boe.es/eli/es/rd/2017/02/24/124). No ABS permits were granted as utilization of the material falls under exemption in Article 3(2) of the Spanish Royal Decree 124/2017 and was confirmed by the national authorities. The whole dry individual was harvested from the tree and was taken alive directly to the laboratory. Dry apical parts of the gametophores with double checked taxonomic identification were individually selected, eliminating sporophytes and reducing contamination risks, carefully manipulating the plant to avoid modifying gene expression. Shots were then weighed and preserved at –80 °C until DNA extraction.

### Vouchering information

The colony was deposited in the herbarium of the Universidad Autónoma de Madrid https://sweetgum.nybg.org/science/ih/herbarium-details/?irn=172673 under voucher ID MAUAM-Brio 5489.

Frozen reference tissue material is available from the same individual at the Museo Nacional de Ciencias Naturales https://www.mncn.csic.es/es under voucher ID MNCN-ADN 151724.

### Genetic information

The estimated genome size, based on ancestral taxa, is 0.48 Gb. This is a diploid genome with a haploid number of 6 chromosomes (2n=12). All information for this species was retrieved from Genomes on a Tree ( Challis *et al.*, 2023).

### DNA/RNA processing

DNA was extracted from approximately 200 mg of modular colony tissue ground in liquid nitrogen with mortar and pestle. The resulting powder was incubated in 10 mL of extraction buffer (100 mM Tris–HCl, 20 mM EDTA, 1.5 M NaCl, 1% sarkosyl, 2% polyvinylpyrrolidone [PVP], and 0.2% β-mercaptoethanol) at 21 °C for 1 h under agitation (300 rpm). DNA purification was subsequently performed following the general procedure described by Ramakrishnan *et al.*, 2017. DNA fragment size selection was performed using Short Read Eliminator (PacBio). DNA quantification was performed using a Qubit dsDNA BR Assay Kit (Thermo Fisher Scientific), and DNA integrity was assessed using a Genomic DNA 165 Kb Kit (Agilent) on the Femto Pulse system (Agilent). The DNA was stored at +4ºC until used.

RNA was extracted from a modular colony (50 mg) using RNeasy Powerplant Kit (Qiagen) following manufacturer instructions. Residual genomic DNA was removed with 6U of TURBO DNase (2 U/µl) (Thermo Fisher Scientific). Quantification was performed using a Qubit RNA HS Assay and integrity was assessed in a Bioanalyzer system (Agilent). RNA was stored at -80°C until used.

### Library preparation and sequencing

Long-read DNA library was prepared with SMRTbell prep kit 3.0 following manufacturers' instructions and sequenced on a Revio system (PacBio). Hi-C library was generated from the same colony used for DNA extraction using the Arima High Coverage HiC Kit (following the Animal Tissues low input protocol v01) and sequenced on NovaSeq X Plus instrument (Illumina) with 2x 150 read length. In total 39x HiFi and 150x HiC data were sequenced to generate the assembly.

### Genome assembly methods

The genome was assembled using the Genoscope GALOP pipeline ( https://workflowhub.eu/workflows/1200). Briefly, raw PacBio HiFi reads were assembled using Nextdenovo v2.5.2 ( Hu *et al.*, 2024). Remaining allelic duplications were removed using purge_dups v1.2.5 ( Guan *et al.*, 2020) with default parameters and the proposed cutoffs. This assembly was scaffolded using YaHS v1.2.2 ( Zhou *et al.*, 2023) and assembled scaffolds were then curated through manual inspection using PretextView v0.2.5 ( Harry, 2022) to remove false joins and incorporate sequences not automatically scaffolded into their respective locations within the chromosomal pseudomolecules. Chromosome-scale scaffolds confirmed by Hi-C data were named in order of size. Finally, the mitochondrial and chloroplastic genomes were assembled respectively as a single contig of 104,820 bp and two contigs of 123,029 bp and 122,994 bp using the Oatk v1.0 ( Zhou *et al.*, 2025) and included in the released assembly (GCA_965213355.2). Summary analysis of the released assembly was performed using the ERGA-BGE Genome Report ASM Galaxy workflow ( https://doi.org/10.48546/workflowhub.workflow.1104.1).

## Results

### Genome assembly

**Figure 1.**
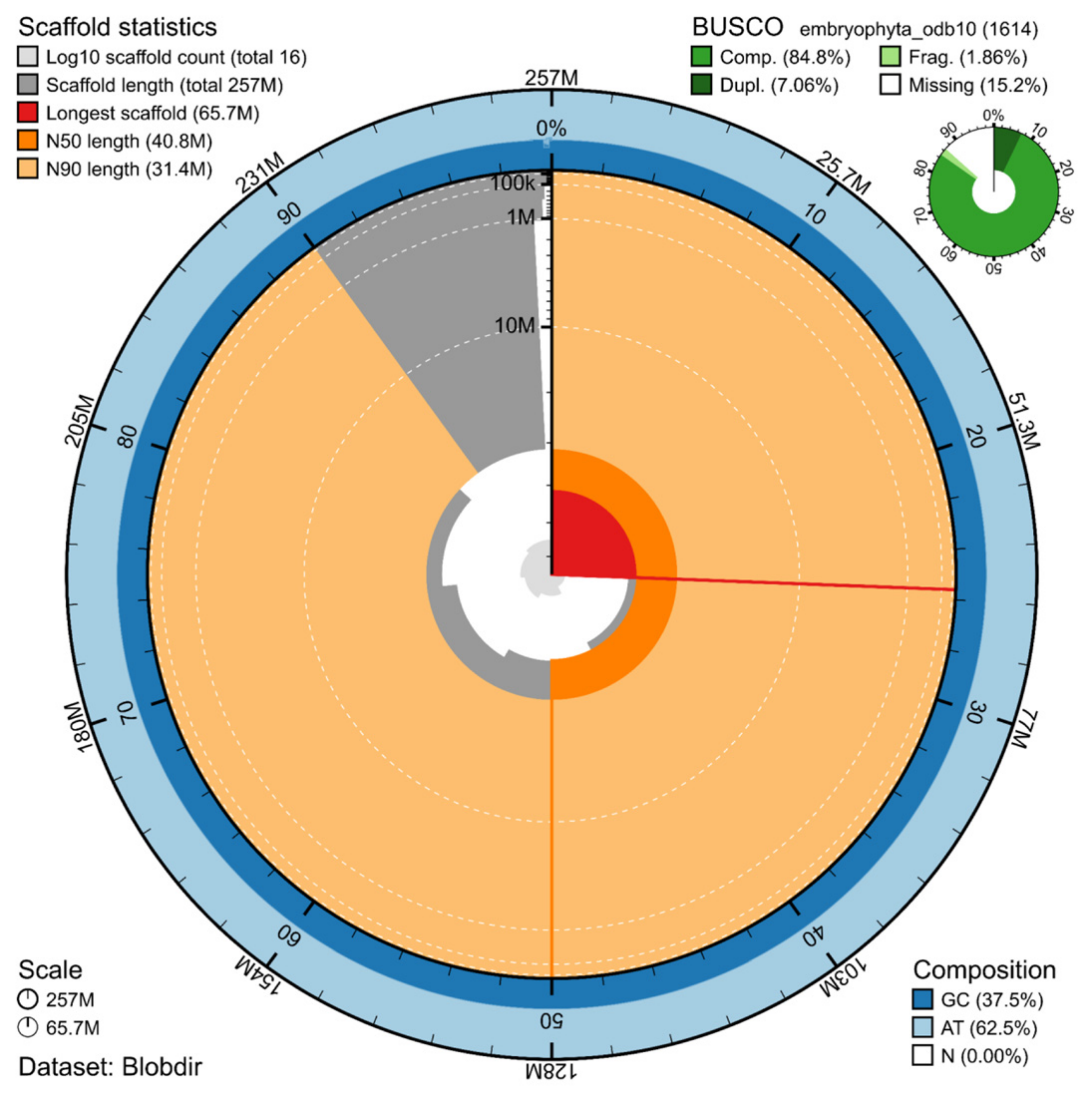
Snail plot summary of assembly statistics. The main plot is divided into 1,000 size-ordered bins around the circumference, with each bin representing 0.1% of the 256,705,263 bp assembly including the mitochondrial genome. The distribution of sequence lengths is shown in dark grey, with the plot radius scaled to the longest sequence present in the assembly (65.7 Mb, shown in red). Orange and pale-orange arcs show the scaffold N50 and N90 sequence lengths (40,771,022 and 31,400,860 bp), respectively. The pale grey spiral shows the cumulative sequence count on a log-scale, with white scale lines showing successive orders of magnitude. The blue and pale-blue area around the outside of the plot shows the distribution of GC, AT, and N percentages in the same bins as the inner plot. A summary of complete, fragmented, duplicated, and missing BUSCO genes found in the assembled genome from the Embryophyta database (odb10) is shown in the top right.

**Figure 2.**
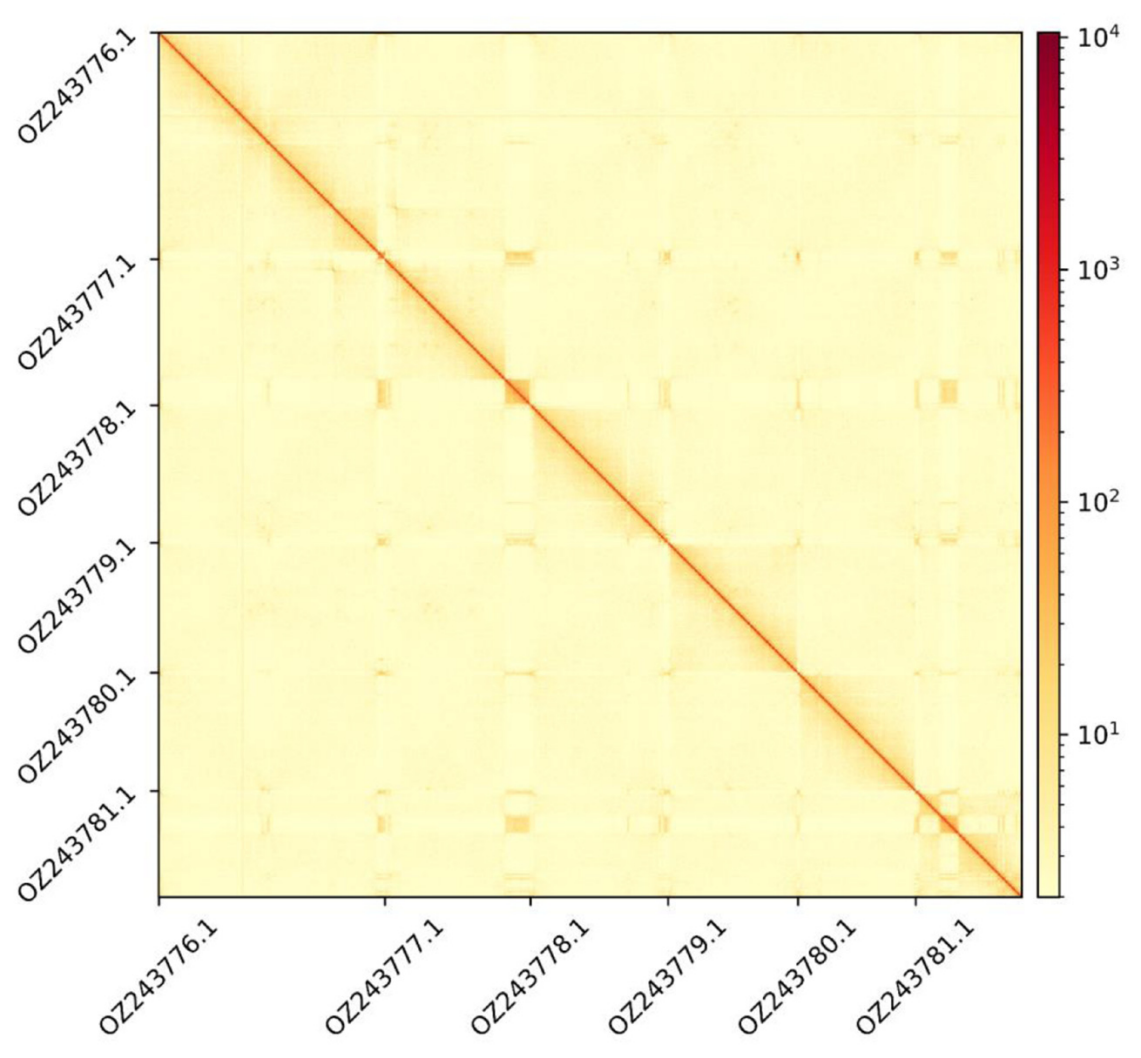
Hi-C contact map showing spatial interactions between regions of the genome. The diagonal corresponds to intra-chromosomal contacts, depicting chromosome boundaries. The frequency of contacts is shown on a logarithmic heatmap scale. Hi-C matrix bins were merged into a 150 kb bin size for plotting.

The genome assembly has a total length of 256,705,263 bp in 13 scaffolds including the mitogenome ( Figure 1 & Figure 2), with a GC content of 37.5%. The assembly has a contig N50 of 11,461,491 bp and L50 of 7 and a scaffold N50 of 40,771,022 bp and L50 of 3. The assembly has a total of 38 gaps, totalling 4.7 kb in cumulative size. The single-copy gene content analysis using the Embryophyta database with BUSCO ( Manni *et al.*, 2021) resulted in 84.8% completeness (77.7% single and 7.1% duplicated). 97.5% of reads k-mers were present in the assembly and the assembly has a base accuracy Quality Value (QV) of 54.4 as calculated by Merqury ( Rhie *et al.*, 2020).

## Data Availability

*Lewinskya acuminata* and the related genomic study were assigned to Tree of Life ID (ToLID) 'cbLewAcum8' and all sample, sequence, and assembly information are available under the umbrella BioProject PRJEB79984. The sample information is available at the following BioSample accessions: SAMEA115283941 and SAMEA115283947. The genome assembly is accessible from ENA under accession number GCA_965213355.2 and the annotated genome will be available through the Ensembl webpage ( https://projects.ensembl.org/erga-bge/). Sequencing data produced as part of this project are available from ENA at the following accessions: ERX14096377, ERX14170726, and ERX14514454. Documentation related to the genome assembly and curation can be found in the ERGA Assembly Report (EAR) document available at https://github.com/ERGA-consortium/EARs/tree/main/Assembly_Reports/Lewinskya_acuminata/cbLewAcum8. Further details and data about the project are hosted on the ERGA portal at https://portal.ergbiodiversity.eu/data_portal/1172132.

## Author information

Members of the Genoscope Sequencing Team are listed here: https://zenodo.org/records/14611490.
