# Supplementary material for: ERGA-BGE reference genome of Lewinskya acuminata, a common epiphytic Mediterranean moss with disjunct populations in California and Ethiopia
Source: Open Res Eur. 2026 Mar 30;5:357. Originally published 2025 Nov 24. [Version 2] doi: 10.12688/openreseurope.21670.2 (PMC12873536; doi:10.12688/openreseurope.21670.2)
Supplement: Peer review history

# Reviewer report: Reviewer response for version 2

**DOI:** 10.21956/openreseurope.24883.r71731

**Published:** 2026-04-08

**Authors:** Panagiotis Ioannidis

**Affiliations:** 1 Foundation for Research & Technology- Hellas, Crete, Greece

The authors have addressed all my concerns and I have no further comments.

Are sufficient details of methods and materials provided to allow replication by others?

Yes

Is the rationale for creating the dataset(s) clearly described?

Yes

Are the datasets clearly presented in a useable and accessible format?

Yes

Are the protocols appropriate and is the work technically sound?

Yes

Reviewer Expertise:

insect genomics; bioinformatics

I confirm that I have read this submission and believe that I have an appropriate level of expertise to confirm that it is of an acceptable scientific standard.

# Reviewer report: Reviewer response for version 1

**DOI:** 10.21956/openreseurope.23442.r67496

**Published:** 2026-02-04

**Authors:** Annabel Whibley

**Affiliations:** 1 Grapevine Improvement, Bragato Research Institute, Lincoln, New Zealand

The authors report the assembly of the genome a reference individual of the epiphytic moss *Lewinskya acuminata*. Good coverage of HiFi reads and Illumina HiC data were combined to produce an assembly that could be scaffolded into 6 pseudochromosomes (the expectation given 2n =12) and a small number of unplaced contigs, plus the organelles.

 In general, the assembly stats are very good, but the BUSCO reports relatively low completeness (84%) and quite high duplication (7%) which might suggest some underlying complications.  It could be that this taxonomic group is not well-catered for by the ODB datasets, but that is not my understanding for bryophytes in general. Some comment on this would be valuable.

 The assembly pipeline makes no direct mention of polishing (although I note that polishing options are available in the Galop pipeline). Could this be made more clear?  There are some omissions from the reporting that I would like to see included and the template is less comprehensive and more unstructured than the standard DToL reporting.  This would be resolved with better templating and a more comprehensive set of metadata in tables (What Embryophyta gene set version is being assessed, for example?).

 I note that a voucher specimen was retained but wonder whether verification of species via DNA barcode might also be a useful step in the sample QC.

 Personally, I would like to see some information on the properties of the gDNA since the authors have performed extensive QC of the sample. I would also prefer to see some information on the kmer profiling (does genome size/heterozygosity fit with expectations?). RNA has been extracted but an annotation is not provided in this report.

 The two reported chloroplast assemblies obtained are colinear and are 99.89% identical.  I would like to see additional supporting evidence that this is not an assembly artefact.

 I would also query the extent to which it is useful to contain both chloroplast sequences in the reference assembly if this represents genuine heteroplasmy- with this degree of similarity it may be more helpful to map to a shared reference and call variants.

 The confusion in total scaffold number (13 in the text vs 16 in the snail plot) I think reflects the inclusion of the organelle genomes in the blobtools analysis- perhaps this could be clarified.

 Please note that Kb should be kb (e.g. in  Genomic DNA 165 Kb Kit).

Are sufficient details of methods and materials provided to allow replication by others?

Yes

Is the rationale for creating the dataset(s) clearly described?

Yes

Are the datasets clearly presented in a useable and accessible format?

Yes

Are the protocols appropriate and is the work technically sound?

Partly

Reviewer Expertise:

Genomics, Bioinformatics, Evolution

I confirm that I have read this submission and believe that I have an appropriate level of expertise to confirm that it is of an acceptable scientific standard, however I have significant reservations, as outlined above.

# Response

**Published:** 2026-02-05

**Authors:** Chiara Bortoluzzi

**Affiliations:** Swiss Institute of Bioinformatics, Lausanne, Vaud, Switzerland

We thank the reviewer for their comment. We addressed all comments as follow: 

1. Also pointed out by the other reviewers, we now clarified in the main text why the BUSCO completeness score is relatively low. Briefly, the high missing score is often elevated when using the embryophyta_odb10 lineage set in bryophytes, as the lineage is biased towards vascular plants.
2. We have expanded the presentation of the results, as also suggested by another reviewer. The Report now has an additional Table on the set of chromosomes and their length and an additional figure (blob plot).
3. No verification of species via DNA barcode was performed. 
4. Most of the information requested by the reviewer (k-mer, heterozygosity, etc) is already reported in the ERGA Assembly Report or EAR, which is reported in the main text already. RNA was extracted, but the annotation is not provided because is undergoing.
5. As also suggested by another reviewer, we now corrected the confusion associated with the number of chromosomes.  

# Reviewer report: Reviewer response for version 1

**DOI:** 10.21956/openreseurope.23442.r67500

**Published:** 2026-01-20

**Authors:** Panagiotis Ioannidis

**Affiliations:** 1 Foundation for Research & Technology- Hellas, Crete, Greece

This manuscript describes the sequencing and assembly of the genome of the plant species Lewinskya acuminata. The Introduction section includes enough general details about this species, mostly regarding its habitat and ecology.

 Additionally, the Methods section provides a detailed account of the sequencing and assembling methodology. Most of the computational tools used for assembling are also widely used in other genome sequencing projects (purge_dups, YaHS, PretextView).

 The resulting genome assembly is very contiguous with a relatively low CC ratio (13 scaffolds / 6 chromosomes = 2.16). Moreover, its QV score (54.4) and k-mer completeness (97.5%) are good. However, its BUSCO score (84% complete BUSCOs) denotes that this assembly could possibly be better; most of the HiFi-based assemblies these days have a BUSCO score of >97%. I would strongly recommend that the authors try another genome assembler such as Hifiasm which is known to produce high quality genome assemblies from HiFi reads.

 I was also happy to see that a genome annotation will be also available for this assembly (using the herein generated RNAseq data, I'm assuming?). This is very important as it drastically increases the usefulness of a genome assembly; without one, a genome assembly cannot be used for quite a number of comparative studies. Unless, of course, genome annotation is performed which is a resource-demanding technically-challenging process.

 Additionally, I believe that there are a few things missing from the Results section, which would greatly help in evaluating the quality of the assembly

 1) some sort on QC on the HiFi reads would be necessary; for example, a GenomeScope plot. This is essential for showing the quality of your starting material.

 2) a table is needed in which to display all the basic assembly features (contig N50, scaffold N50, L50, BUSCO scores, number of contigs, number of scaffolds etc).

 3) another table is needed showing the lengths (and accessions) of the chromosome-level scaffolds so that the reader knows what the exact length of each chromosome.

 4) a blob plot is also necessary in order to get a proper overview of the assembly scaffolds. I find this plot especially useful for many reasons; for checking the GC content and sequencing coverage of the assembly scaffolds, as well as for getting a hint about the possible origin of the extra scaffolds.

Are sufficient details of methods and materials provided to allow replication by others?

Yes

Is the rationale for creating the dataset(s) clearly described?

Yes

Are the datasets clearly presented in a useable and accessible format?

Yes

Are the protocols appropriate and is the work technically sound?

Yes

Reviewer Expertise:

insect genomics; bioinformatics

I confirm that I have read this submission and believe that I have an appropriate level of expertise to confirm that it is of an acceptable scientific standard, however I have significant reservations, as outlined above.

# Response

**Published:** 2026-01-27

**Authors:** Chiara Bortoluzzi

**Affiliations:** Swiss Institute of Bioinformatics, Lausanne, Vaud, Switzerland

We thank the reviewer for their comments and suggestions. All comments have been addressed as follow:

1. As also pointed out by Reviewer 1, the 'low' BUSCO completeness score is due to a bias in the embryophyta database used in the BUSCO analysis. We described this bias in the 'Results' section.
2. Yes, the genome annotation will be available through the Ensembl website and it will make use of the RNA-Seq data generated along with the genome assembly.
3. All these additional QC are already reported in the ERGA Assembly Report (or EAR) which accompanies this Genome Report, as reported in the Methods section ( https://github.com/ERGA-consortium/EARs/blob/main/Assembly_Reports/Lewinskya_acuminata/cbLewAcum8/cbLewAcum8.1_EAR.pdf). 
4. As before, all these additional information are already reported in the EAR ( https://github.com/ERGA-consortium/EARs/blob/main/Assembly_Reports/Lewinskya_acuminata/cbLewAcum8/cbLewAcum8.1_EAR.pdf). 
5. We added a table showing the lenghts (and accessions) of the chromosome-level assembly. 
6. We added a Figure showing the blob plot results.

# Response

**Published:** 2026-01-30

**Authors:** Chiara Bortoluzzi

**Affiliations:** Swiss Institute of Bioinformatics, Lausanne, Vaud, Switzerland

We thank the reviewer for their feedback. We have addressed all commens as follow:

1. As also pointed out by Reviewer 1, the ‘low’ BUSCO completeness score is due to a bias in the embryophyta database used in the BUSCO analysis. We described this bias in the Results section.
2. Yes, the genome annotation will be available through the Ensembl website and it will make use of the RNA-Seq data generated along with the genome assembly.
3. All these additional QC are already reported in the ERGA Assembly Report (or EAR) which accompanies this Genome Report, as reported in the Methods section ( https://github.com/ERGA-consortium/EARs/blob/main/Assembly_Reports/Lewinskya_acuminata/cbLewAcum8/cbLewAcum8.1_EAR.pdf).
4. We added an additional table to the Report with information on the chromosomes and their length.
5. We now added a new Figure (blobl plot) to address this comment.

# Reviewer report: Reviewer response for version 1

**DOI:** 10.21956/openreseurope.23442.r65418

**Published:** 2026-01-02

**Authors:** Qilin Yang

**Affiliations:** 1 University of Chinese Academy of Sciences, Beijing, China

This Genome Note presents a high-quality reference genome assembly for the epiphytic moss Lewinskya acuminata (Orthotrichaceae). The resulting assembly is highly contiguous, resolving into 6 chromosomal pseudomolecules (consistent with the known karyotype) with a total length of ~257 Mb and high base quality (QV >50). The authors clearly outline the significance of the species for phylogenomic and biogeographical studies within the Mediterranean and disjunct populations.

 General Comments

 The manuscript is well-written, the methodology is state-of-the-art, and the resulting data appears to be of excellent quality. The Hi-C map (Figure 2) shows very clean chromosome separation, and the Snail plot (Figure 1) indicates high contiguity. The work is technically sound and provides a valuable resource for the bryology and genomics communities.

 I have answered "Yes" to all questionnaire items, as the data is accessible and the methods are replicable. However, I have a few specific suggestions for clarification regarding the description of ploidy/genome size and the organelle assembly that would improve the precision of the manuscript before final indexing.

 Specific Points for Improvement:

 1. Clarification of Ploidy and Life Stage

 In the "Genetic information" section, the text states: "The estimated genome size, based on ancestral taxa, is 0.48 Gb. This is a diploid genome with a haploid number of 6 chromosomes (2n=12)."

 Later, in "Sample and sampling information," the authors correctly note they sampled "apical parts of the gametophores," which in mosses represents the haploid (n) life stage.

 The final assembly size is ~0.25 Gb. This is perfectly consistent (half the size) with the 0.48 Gb estimate if the estimate refers to the 2C (diploid) content and the assembly represents the 1C (haploid) content.

 Suggestion: Please clarify the "Genetic information" section to explicitly state that the target assembly is the haploid genome (n=6). Phrasing it as "This is a diploid genome" is technically true for the sporophyte phase but potentially confusing for a gametophyte assembly. Explicitly stating that the assembled size (0.25 Gb) aligns with the haploid expectation derived from the 0.48 Gb diploid estimate would assist the reader.

 2. Plastid Genome Description

 In the Abstract, it is stated: "...1 mitochondrial genome, and 2 plastid genomes."

 In the Methods: "...chloroplastic genomes were assembled respectively as ... two contigs of 123,029 bp and 122,994 bp..."

 Suggestion: It is highly unusual for a moss to have two distinct plastid genomes. It is more likely that the assembly process resolved the plastome into two contigs (perhaps split by the Inverted Repeat regions) or that the software output two isomers.

 If these are two contigs representing one plastid genome, please rephrase to "the plastid genome was assembled into 2 contigs."

 If these represent two structural isomers of the same genome, please clarify this to avoid the impression that the species possesses two biological plastid genomes.

 3. BUSCO Scores

 The BUSCO completeness is 84.8% (Missing 15.2%). While this is a good score, "Missing" scores in bryophytes are often elevated when using the standard embryophyta_odb10 lineage set, as the dataset is biased towards vascular plants.

 Suggestion: A brief sentence in the Results acknowledging that the missing BUSCO percentage is typical for bryophytes assessed against the Embryophyta dataset would provide better context for non-specialist readers who might otherwise view >10% missing genes as a quality issue.

 4. Scaffold Count Discrepancy

 Text: "The genome assembly has a total length of 256,705,263 bp in 13 scaffolds..."

 Figure 1 Legend: "Log10 scaffold count (total 16)"

 Suggestion: Please reconcile these numbers. It is possible the Snail plot includes small unlocalized contigs or the organelles that were not counted in the text summary.

 5. Typo

 Abstract: "...six contiguous chromosomal pseudomolecules..."

 Results: "...contig N50 of 11.5 Mb..."

 The text is generally clean, but please ensure the distinction between "contigs" and "scaffolds" is used consistently when discussing the final chromosome-level assembly. (The current usage seems correct, this is just a reminder to double-check).

Are sufficient details of methods and materials provided to allow replication by others?

Yes

Is the rationale for creating the dataset(s) clearly described?

Yes

Are the datasets clearly presented in a useable and accessible format?

Yes

Are the protocols appropriate and is the work technically sound?

Yes

Reviewer Expertise:

plant_molecular_biology_and_bioinformatics

I confirm that I have read this submission and believe that I have an appropriate level of expertise to confirm that it is of an acceptable scientific standard.

# Response

**Published:** 2026-01-27

**Authors:** Chiara Bortoluzzi

**Affiliations:** Swiss Institute of Bioinformatics, Lausanne, Vaud, Switzerland

We thank the reviewer for their comments and for their kind words. We have addressed all points as follow: 

 1. We have now corrected the 'Genetic information' section as suggested.

 2. We have changed the abstract to match what reported in the 'Methods' section to avoid any further confusion.

 3. We thank the reviewer for point out the issue with the BUSCO lineage. We have now corrected this in the 'Results' section to provide more context.

 4. We have now corrected Figure 1. 

 5. All typos have been corrected.

# Response

**Published:** 2026-01-30

**Authors:** Chiara Bortoluzzi

**Affiliations:** Swiss Institute of Bioinformatics, Lausanne, Vaud, Switzerland

We thank the reviewer for their comments. We have addressed all comments as follow:

1. We now corrected the ‘Genetic information’ section as suggested.
2. We changed the abstract to match what reported in the Methods section to avoid any further confusion.
3. We have corrected the BUSCO score in the Results section. 
4. We have now corrected Figure 1.
5. We have corrected all typos.
